# TAK1 mediates excessive autophagy via p38 and ERK in cisplatin‐induced acute kidney injury

**DOI:** 10.1111/jcmm.13585

**Published:** 2018-03-05

**Authors:** Jun Zhou, Youling Fan, Jiying Zhong, Zhenxing Huang, Teng Huang, Sen Lin, Hongtao Chen

**Affiliations:** ^1^ Department of Anesthesiology The First People's Hospital of Foshan Foshan China; ^2^ Department of Anesthesiology Panyu Central Hospital Guangzhou China; ^3^ Department of Anesthesiology Eighth People's Hospital of Guangzhou Guangzhou China

**Keywords:** acute kidney injury, autophagy, ERK, p38, TAK1

## Abstract

The ability of cisplatin (cis‐diamminedichloroplatinum II) toxicity to induce acute kidney injury (AKI) has attracted people's attention and concern for a long time, but its molecular mechanisms are still widely unknown. We found that the expression of transforming growth factor‐β (TGF‐β)‐activated kinase 1 (TAK1) could be increased in kidneys of mice administrated with cisplatin. Autophagy is an evolutionarily conserved catabolic pathway and is involved in various acute and chronic injuries. Moreover, p38 MAPK (mitogen‐activated protein kinase) and ERK regulate autophagy in response to various stimuli. Therefore, our hypothesis is that cisplatin activates TAK1, which phosphorylates p38 and ERK, leading to excessive autophagy of tubular epithelial cells and thus exacerbating kidney damage. Here, BALB/c mice were intraperitoneally injected with a TAK1 inhibitor and were then administrated with sham or cisplatin at 20 mg/kg by intraperitoneal injection. Compared with mice in the vehicle cisplatin group, mice intraperitoneally injected with a TAK1 inhibitor were found to have lower serum creatinine and less tubular damage following cisplatin‐induced AKI. Furthermore, inhibition of TAK1 reduced p38 and Erk phosphorylation, decreased expression of LC3II and reversed the down‐regulation of P62 expression induced by cisplatin. The hypothesis was verified with tubular epithelial cells administrated with cisplatin in vitro. Finally, p38 inhibitor or ERK inhibitor abated autophagy activation and cell viability reduction in tubular epithelial cells treated with cisplatin plus TAK1 overexpression vector. Taken together, our results show that cisplatin activates TAK1, which phosphorylates p38 and ERK, leading to excessive autophagy of tubular epithelial cells that exacerbates kidney damage.

## 1 INTRODUCTION

Cisplatin (cis‐diamminedichloroplatinum II), as a class of cytotoxic agents, has been widely used for chemotherapy against tumours. The antitumour and toxic effects of the drug are frequently discussed.^1^ Nephrotoxicity is the most common side effect of the drug's therapeutic effectiveness and is associated with high mortality.^2^ However, the mechanism of cisplatin‐induced AKI remains unclear. A better understanding of the molecular mechanisms underlying cisplatin‐induced AKI is essential to improve the life quality of cancer patients receiving cisplatin chemotherapy.

Autophagy is a highly conservative cell behaviour to maintain intracellular homeostasis and has largely entered the research spotlight only recently.^3^ Autophagy may play a pro‐death or a pro‐survival role of cells.^4^ A large amount of research have shown that autophagy is a double‐edged sword involved in health and disease.^5^ Whether autophagy protects or aggravates the renal damage in cisplatin‐induced AKI is unclear.

Transforming growth factor‐β (TGF‐β)‐activated kinase 1 (TAK1) is a serine/threonine kinase that plays a key role in regulating immune and intracellular signalling pathways.^6^ It has been reported that TAK1 participates in regulatory mechanisms of acute injury in several tissue types.^7^ TAK1 has also been implicated in oxidative stress, apoptosis and autophagy.^8^ However, the role of TAK1 in response to cisplatin‐induced AKI has not been investigated. Moreover, p38 and ERK, as TAK1 downstream kinases,^9^ have recently been implied as involved in autophagy.^10^ It has been reported that p38 MAPK signalling pathway was found to regulate Beclin 1 S90 phosphorylation that is essential for autophagy.^11^ Activation of p38 MAPK pathway regulates the transcription of autophagy genes in response to oxidative stress.^12^ ERK1/2 can phosphorylate G interacting protein (GAIP) and stimulate autophagy.^13^ The results with Szu‐ying Chen suggested the necessity of ERK for autophagic cell death.^14^Therefore, this study aimed to investigate if cisplatin activates TAK1, which phosphorylates p38 and ERK, leading to excessive autophagy of tubular epithelial cells that exacerbates kidney damage in cisplatin‐induced AKI.

## 2 METHODS

### 2.1 Animals

The animal experiments were conducted according to the guidelines of laboratory animal care and were approved by the Institutional Animal Care and Use Committee of the First People's Hospital of Foshan. Cisplatin was dissolved directly in 0.9% saline at 1 mg/mL. Male BALB/c mice, 8‐12 weeks old, were administrated with cisplatin (20 mg/kg) or saline by i.p. injection. TAK1 inhibitor (5Z‐7‐oxozeaenol) (Sigma‐Aldrich, Rehovot, Israel) 4 mg/kg and an equal volume of 0.9% normal saline were i.p. injected into the TAK1 inhibitor group and vehicle group mice, respectively. The first injection of TAK1 inhibitor or saline was 1 hour before injection of cisplatin or sham control, once per day for 3 days. The first injection of 3‐MA was 1 hour before injection of cisplatin or sham control (20 mg/kg/d, i.p.). Animals were killed at 72 hours after cisplatin injection. Kidneys were perfused and harvested.

### 2.2 Measurement of renal function

Serum creatinine was measured using a creatinine assay kit (BioAssay Systems, Hayward, CA) according to the manufacturer's instructions. Blood urea nitrogen was determined fluorometrically as described.^15^

### 2.3 Renal morphology

Kidney tissue was fixed in 10% buffered formalin, embedded in paraffin and cut at 4‐μm thickness. After deparaffinization and rehydration, sections were stained with periodic acid‐Schiff (PAS). Tissue damage was examined in a blinded manner and scored as reported in a previous study.^15^

### 2.4 Immunohistochemistry

Immunohistochemical staining was performed on paraffin sections. Antigen retrieval was performed with antigen unmasking solution (Vector Laboratories, Burlingame, CA). Slides were incubated with the primary antibody (TAK1, Abcam, Cambridge, UK) and appropriate secondary antibody for a suitable period of time after blocking. Immunohistochemical staining was performed with the avidin‐biotin complex (ABC) method according to the protocol of the Vector ABC kit (Vector Laboratories, Burlingame, CA). The images from these slides were acquired and analysed by NIS Element software with a Nikon microscope imaging system.

### 2.5 Western blot analysis

Protein was extracted using RIPA buffer containing proteinase inhibitor cocktail and quantified with a Bio‐Rad protein assay. An equal amount of protein was separated on SDS‐polyacrylamide gels in a Tris/SDS buffer system and then transferred onto nitrocellulose membranes. Blotting was performed according to standard procedures with primary antibodies against LC3 I/II,P62, p38 and ERK overnight, followed by incubation with appropriate fluorescence‐conjugated secondary antibodies. The proteins of interest were analysed using an Odyssey IR scanner, and signal intensities were quantified using NIH Image/J software.^15^

### 2.6 Cell culture and treatment

Mouse renal tubular epithelial cells (EpiCM‐a)(Sciencell, San Diego, California, US) were thawed in a 38°C water bath and then centrifuged at 200 g for 5 minutes. The supernatant was discarded, and the cells were cultured in DMEM medium in an incubator containing 5% CO_2_ at 37°C. The medium was replaced when cells adhered to the bottle wall. The cells were subcultured until the cells covered 80% of the bottom of the bottle. Cells were pre‐treated for 1 hours with 5Z‐7‐oxozeaenol dissolved in dimethyl sulfoxide (10 μmol/L in DMSO) or 3‐MA (10 μmol/L) according to the manufacturer's recommendation. Cells were treated with 10 μg/mL cisplatin or sham control for 6 hours.

### 2.7 Cell transfection with TAK1 overexpression plasmid or TAK1 siRNA

The overexpression plasmid of the TAK1 gene was manufactured by Mingshanshang Medical Biotechnology Co., Ltd. (Guangzhou City, P.R. China). It was verified via polymerase chain reaction (PCR), restriction enzyme digestion and DNA sequencing. TAK1 siRAN were purchased from Santa Cruz Biotechnology (Santa Cruz Biotechnologies, Santa Cruz, CA, USA). Suspension cells: just prior to preparing complexes, 4‐8 × 10^5^ cells were plated in 500 μL of growth medium without antibiotics. Each transfection sample was performed using LipofectamineTM 2000 according to the manufacturer's instructions. 500 ng of DNA was transfected as for pSELECT‐GFP‐LC3 plasmid 24 hours. Cells were incubated at 37°C in a CO_2_ incubator prior to treatment with cisplatin. Medium was changed after 4‐6 hours.

### 2.8 Quantitative real‐time RT‐PCR

Total RNA was extracted from kidney tissues with TRIzol reagent (Invitrogen). Aliquots (1 μg) of total RNA were reverse‐transcribed using SuperScript II reverse transcriptase. Real‐time PCR was performed using an IQ SYBR green supermix reagent (Bio‐Rad, Herculus, CA) with a Bio‐Rad real‐time PCR machine according to the manufacturer's instructions. The comparative Ct method (ΔΔCt) was used to quantify gene expression, and the relative quantification was calculated as 2^−ΔΔCt^. The expression levels of the target genes were normalized to GAPDH levels in each sample. The primer sequences were as follows: TAK1‐forward, 5′‐TGGACGTTTAAGCTTGGGAGC‐3′, ‐reverse, 5′‐CCAGTTCTGCAACTAGTTCTTGC‐3′; GAPDH‐forward, 5′‐CCAATGTGTCCGTCGCGTGGATCT‐3′, ‐reverse, 5′‐GTTGAAGTCGCAGGAGACAACC‐3′.

### 2.9 Intracellular LC3 fluorescent spot detection

The levels of autophagy were observed with intracellular LC3 fluorescence blobs by the pSELECT‐GFP‐LC3 plasmid. The renal tubule epithelial cells were cultured at a density of 1 × 10^5^ cells/hole, and then the lipofectamine2000 reagent (the Invitrogen, Carlsbad, CA, USA) was used to transfer 500 ng pSELECT‐GFP‐LC3 plasmid to every hole. After 24 hours of transfection, cells were treated, respectively, with 5Z‐7‐oxozeaenol (10 μmol/L), TAK1 overexpression plasmid, TAK1 overexpression plasmid combinated with p38 inhibitor (10 μmol/L, SB203580, Sigma‐Aldrich, St Louis, MO) or TAK1 overexpression plasmid combinated with ERK inhibitor (25 μmol/L, PD98059, Merck Millipore, Darmstadt GE) and then treated with 10 μg/mL cisplatin or sham control 24 hours. The LC3‐GFP fluorescence spots in cells were observed and photographed by fluorescence microscopy (Nikon, Tokyo, Japan).

### 2.10 MTT assay

Cell viability was detected with the MTT assay, a quantitative colorimetric assay with 3‐(4, 5‐dimethylthiazol‐2‐yl)‐2, 5‐diphenyltetrazolium bromide. Cells transfected with TAK1 overexpression plasmid were seeded into 96‐well plates with an appropriate incubation period, and then pre‐treated with 1 μL SB203580 (10 μmol/L in DMSO) or PD98059 (25 μmol/L in DMSO), or DMSO as a control 1 hours prior to exposed to 10 μg/mL cisplatin or sham control for 24 hours. After treatment, 20 μL MTT solution (5 mg/mL, Biotopped, China) and 150 μL DMSO were added in sequence to the wells according to the instructions for kit and the absorbance was measured at 490 nm finally.

### 2.11 Statistical analysis

The data are presented as the means ± SEM. The results were statistically analysed using a one‐way or two‐way analysis of variance (ANOVA). Comparisons between two groups were analysed by performed by Student's *t* test. *P* < .05 was considered statistically significant.

## 3 RESULTS

### 3.1 TAK1 expression and cell autophagy in kidneys of mice with cisplatin‐induced AKI

**Figure 1:**
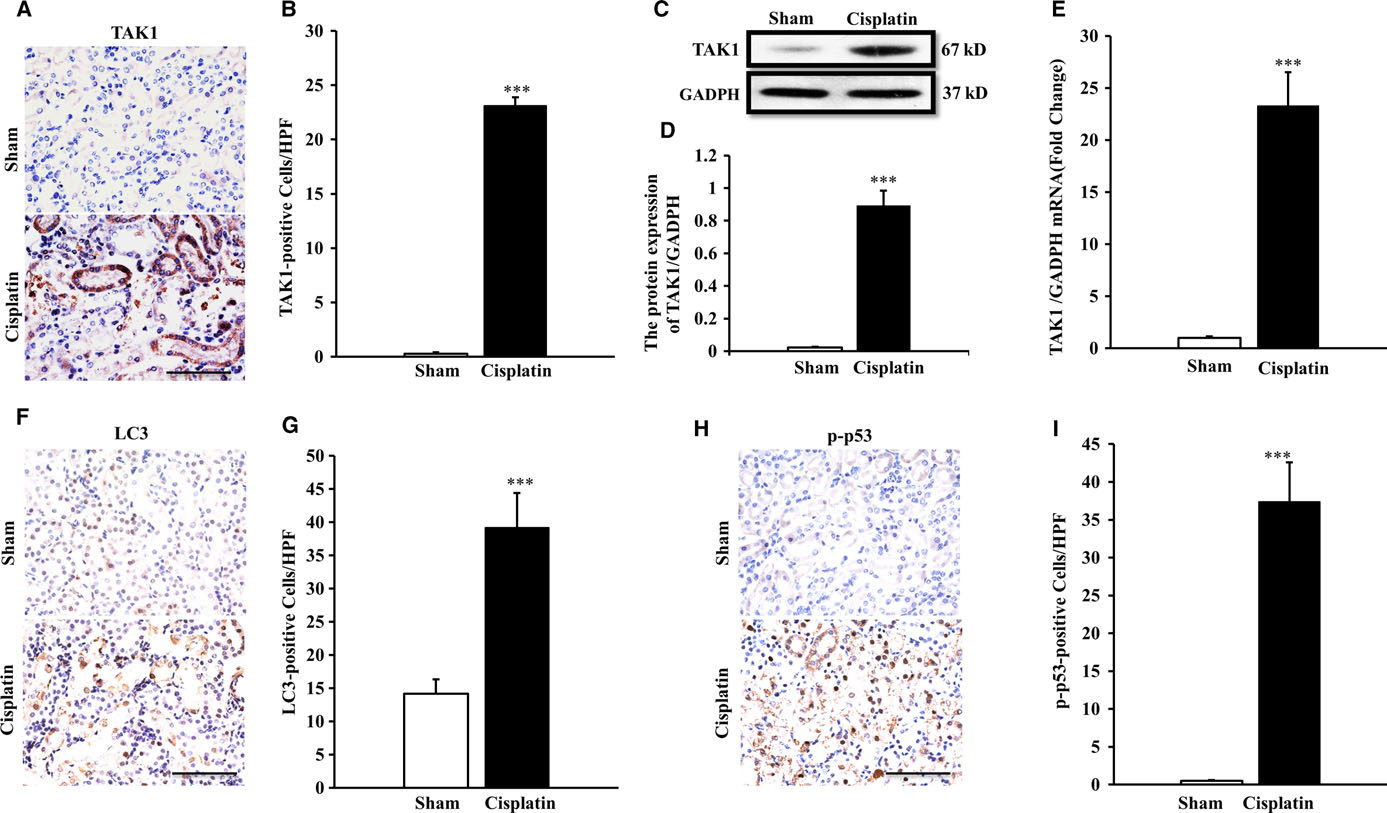
TAK1 expression in kidneys of mice with cisplatin‐induced AKI. A, Representative photomicrographs of TAK1 immunohistochemical staining in kidneys of mice i.p. with cisplatin (20 mg/kg) or saline (Original magnification: ×400, Scale bar:50 μm). B, Quantitative analysis of TAK1‐positive cells in kidneys of mice i.p. with cisplatin (20 mg/kg) or saline. ***P < .001 vs Sham, n = 6 each. C, Representative Western blots show TAK1 protein expression in kidneys of mice after sham or cisplatin treatment. D, Quantitative analysis of protein expression of TAK1 in kidneys of mice. ***P < .001 vs Sham, n = 6 in each group. E, Quantitative analysis of TAK1 mRNA in kidneys of mice. ***P < .001 vs Sham, n = 6 in each group. F, Representative photomicrographs of LC3 immunohistochemical staining in kidneys of mice i.p. with cisplatin (20 mg/kg) or saline (Original magnification: ×400, Scale bar: 50 μm). G, Quantitative analysis of LC3‐positive cells in kidneys of mice. ***P < .001 vs Sham, n = 6 each. H, Representative photomicrographs of p‐p53 immunohistochemical staining in kidneys of mice i.p. with cisplatin (20 mg/kg) or saline (Original magnification: ×400, Scale bar:50 μm). I, Quantitative analysis of p‐p53‐positive cells in kidneys of mice. ***P < .001 vs Sham, n = 6 each

We first investigated whether TAK1 is induced in cisplatin‐induced AKI and where it is expressed in the pathological process. BALB/c mice were treated i.p. with sham or cisplatin at 20 mg/kg, and the number of positive cells assessed by immunohistochemical with TAK1 antibody was counted at 72 hours after cisplatin treatment (Figure 1A). The results revealed that there was a significant increase in the number of TAK1‐positive cells in kidneys of cisplatin‐treated mice compared with sham group mice (Figure 1B). Western blotting analysis using TAK1 antibody showed that the protein expression of TAK1 was significantly higher in kidneys of cisplatin‐treated mice compared with sham group mice (Figure 1C,D). Similarly, real‐time PCR results showed that the expression of TAK1 mRNA was markedly increased in kidneys of mice 72 hours after cisplatin treatment (Figure 1E). Our data indicated that nephrotoxicity of cisplatin induced increased TAK1 expression in renal tubular epithelial cells of mice. LC3 modification is one of the required ubiquitylation‐like modifications during the formation of mammalian autophagosomes. Therefore, the number of positive cells as assessed by immunohistochemical with LC3 antibody was counted at 72 hours after cisplatin treatment (Figure 1F). The results showed that the number of LC3‐positive cells was significantly increased in kidneys of cisplatin‐treated mice compared with sham group mice (Figure 1G). It had been proved that activation of p38 or ERK‐p53 signalling pathway is involved in autophagic cell death and AKI. Hence, we detected the phosphorylation of p53 using IHC method (Figure 1H), and the results showed that p53 was markedly activated in kidneys of mice following cisplatin treatment (Figure 1I). These data prompt that cell autophagy is induced in kidney of mice treated with cisplatin.

### 3.2 Inhibition of TAK1 protects from cisplatin‐induced AKI

**Figure 2:**
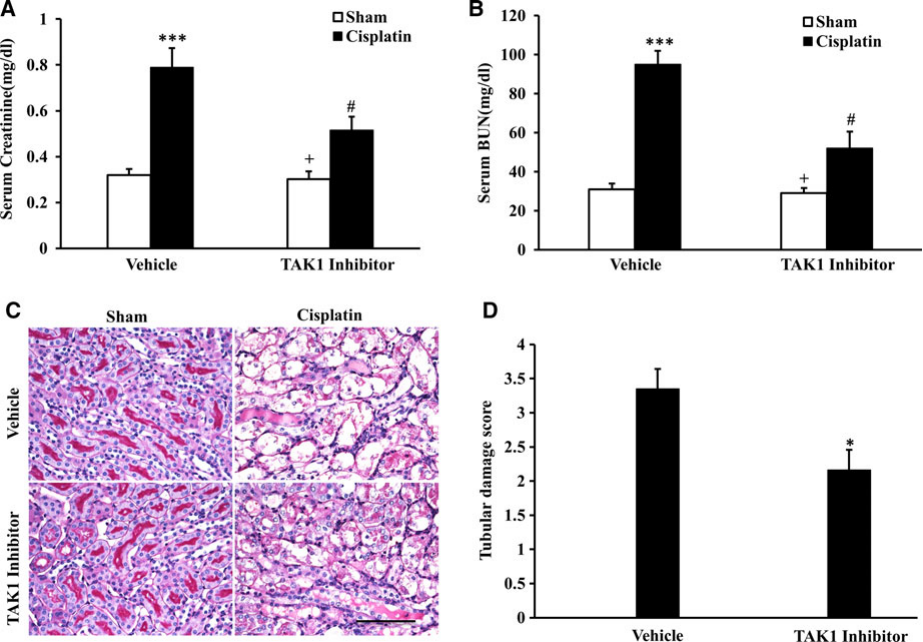
Inhibition of TAK1 protects kidney against cisplatin‐induced AKI. A, Effect of TAK1 inhibition on serum creatinine in vehicle group and TAK1 inhibitor group mice at 72 h after cisplatin or saline treatment. ***P < .001 vs Sham Vehicle; ^+^ *P* < .05 vs TAK1 inhibitor Cisplatin; ^#^ *P* < .05 vs Vehicle Cisplatin. n = 6 in each group. B, Effect of TAK1 inhibition on serum urea nitrogen in Vehicle group and TAK1 inhibitor group mice at 72 h after cisplatin or saline treatment. ***P < .001 vs Sham Vehicle; ^+^ *P* < .05 vs TAK1 inhibitor Cisplatin; ^#^ *P* < .05 vs Vehicle Cisplatin. n = 6 in each group. C, PAS staining for kidney sections of vehicle group and TAK1 inhibitor group mice at 72 h after cisplatin or saline treatment (Original magnification: ×400, Scale bar:50 μm). D, Quantitative assessment of tubular damage in mice at 72 h after cisplatin treatment. *P < .05 vs Vehicle Cisplatin. n = 6 in each group

To investigate the role of TAK1 in the pathogenesis of cisplatin‐induced AKI, a TAK1 inhibitor was used in this study. TAK1 inhibitor or an equal volume of 0.9% normal saline was i.p. injected into mice once per day for 3 days, and intraperitoneal injection of cisplatin was administrated. The results showed that mice developed renal dysfunction, as reflected by marked elevation of blood creatinine and urea nitrogen at 72 hours after cisplatin treatment. Renal function was relatively preserved, with blood creatinine and urea nitrogen being markedly lower in TAK1 inhibitor group mice than vehicle group mice (Figure 2A,B). Consistent with the preservation of kidney function in TAK1 inhibitor group mice following cisplatin treatment, there was a substantial reduction in histological injury of the kidneys, as evidenced by less tubular epithelial cell injury, tubular dilation and intratubular cast formation (Figure 2C,D).

### 3.3 Inhibition of TAK1 against cell autophagy in cisplatin‐induced AKI

**Figure 3:**
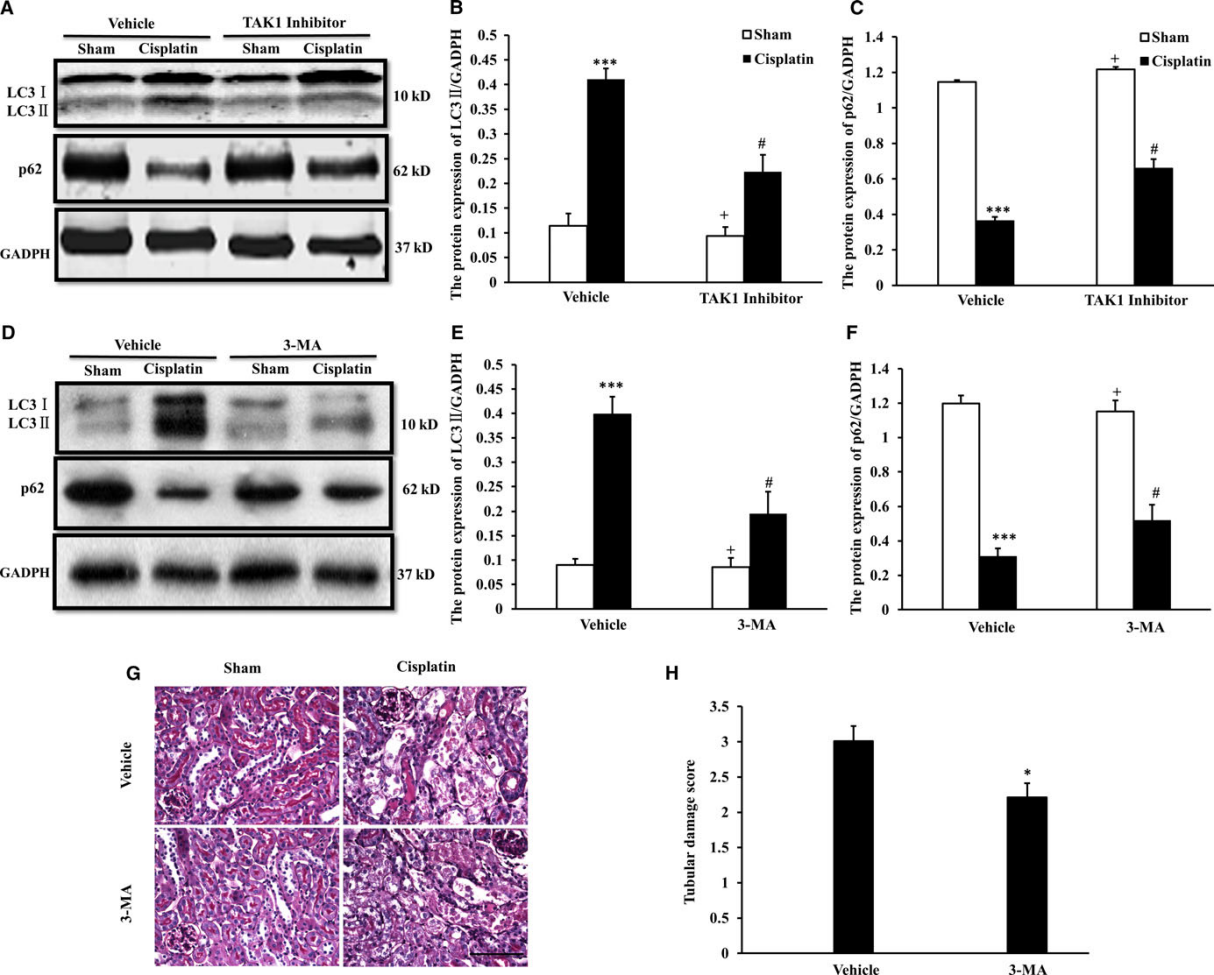
Inhibition of TAK1 decreased autophagy of tubular epithelial cells in cisplatin‐induced AKI. A, Representative Western blots show LC3 I/II and p62 protein expression in kidneys of vehicle group and TAK1 inhibitor group mice (5Z‐7‐oxozeaenol, 4 mg/kg i.p., once per day for 3 d) at 72 h after cisplatin (20 mg/kg) or saline treatment. B, Quantitative analysis of protein expression of LC3II in kidneys of mice. ***P < .001 vs Sham Vehicle; ^+^ *P* < .05 vs TAK1 inhibitor Cisplatin; ^#^ *P* < .05 vs Vehicle Cisplatin. n = 6 in each group. C, Quantitative analysis of protein expression of p62 in kidneys of mice. ***P < .001 vs Sham Vehicle; ^+^ *P* < .05 vs TAK1 inhibitor Cisplatin; ^#^ *P* < .05 vs Vehicle Cisplatin. n = 6 in each group. D, Representative Western blots show LC3 I/II and p62 protein expression in kidneys of vehicle group and 3‐MA group mice (3‐MA, 20 mg/kg/d, i.p., once per day for 3 d) at 72 h after cisplatin (20 mg/kg) or saline treatment. E, Quantitative analysis of protein expression of LC3II in kidneys of mice. ***P < .001 vs Sham Vehicle; ^+^ *P* < .05 vs 3‐MA Cisplatin; ^#^ *P* < .05 vs Vehicle Cisplatin. n = 6 in each group. F, Quantitative analysis of protein expression of p62 in kidneys of mice. ***P < .001 vs Sham Vehicle; ^+^ *P* < .05 vs 3‐MA Cisplatin; ^#^ *P* < .05 vs Vehicle Cisplatin. n = 6 in each group. G, PAS staining for kidney sections of vehicle group and 3‐MA group mice at 72 h after cisplatin or saline treatment (Original magnification: ×400, Scale bar:50 μm). H, Quantitative assessment of tubular damage in mice at 72 h after cisplatin treatment. *P < .05 vs Vehicle Cisplatin. n = 6 in each group

To observe the effect of TAK1 on cell autophagy in the pathogenesis of cisplatin‐induced AKI, we investigated the role of TAK1 in cisplatin‐induced autophagy of tubular epithelial cells in mice treated with a TAK1 inhibitor. LC3 is a central protein in the autophagy pathway, where it functions in autophagy substrate selection and autophagosome biogenesis, which is the most widely used maker of autophagosomes.^16^ The expression level of LC3II represents the level of autophagy. p62, one of the selective substrates for autophagy, interacts with and is degraded alongside polyubiquitinated proteins destined for autophagosomes.^17^ Therefore, p62 protein levels decrease upon autophagy induction.^18^ Western blotting analysis using antibodies against LC3II showed that the expression of LC3II was significantly higher in kidneys of vehicle mice following cisplatin treatment compared with vehicle‐sham mice and p62 protein was markedly decreased in kidneys of vehicle mice following cisplatin treatment (Figure 3A). The expression of LC3II was significantly decreased in kidneys of TAK1 inhibitor i.p. mice after cisplatin treatment compared with vehicle mice treated with cisplatin (Figure 3B). The level of p62 protein was significantly increased in cisplatin‐induced kidneys of TAK1 inhibitor i.p. mice compared with vehicle mice (Figure 3C). Moreover, to complete the analysis of autophagy flux with the index of LC3II and p62, we performed the same experiments in the presence or not autophagy inhibitors in vivo. The methyladenine (3‐MA), as a commonly used autophagy inhibitor, was intraperitoneal injected into mice with cisplatin‐induced AKI model. The change trend of LC3II and p62 in kidneys of 3‐MA i.p. mice with Western blotting analysis was consistent with TAK1 inhibitor i.p. mice (Figure 3D‐F). Then, we determined using 3‐MA if the inhibition of cisplatin‐induced autophagy prevents AKI by analysing histological injury of the kidneys as for TAK1 inhibitor, there was a substantial reduction in histological injury of the kidneys of 3‐MA i.p. mice, as evidenced by less tubular epithelial cell injury, tubular dilation and intratubular cast formation (Figure 3G,H). These data indicate that TAK1 signalling promotes cell autophagy in the kidney in response to cisplatin treatment.

### 3.4 Inhibition of TAK1 decreases phosphorylation of p38 and ERK in kidneys of mice with cisplatin‐induced AKI

**Figure 4:**
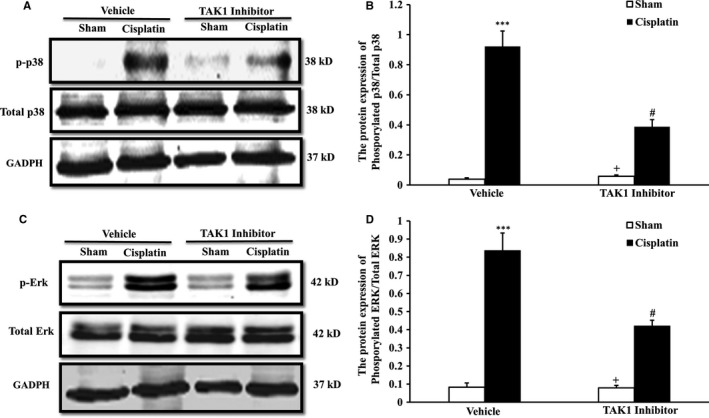
Inhibition of TAK1 decreased phosphorylation of p38 and ERK in kidneys of mice with cisplatin‐induced AKI. A, Representative Western blots show p38 protein expression in kidneys of vehicle group and TAK1 inhibitor group mice (5Z‐7‐oxozeaenol, 4 mg/kg i.p., once per day for 3 d) at 72 h after cisplatin (20 mg/kg) or saline treatment. B, Quantitative analysis of protein expression ratio of phosphorylated p38 and total p38 in kidneys of mice. ***P < .001 vs Sham Vehicle; ^+^ *P* < .05 vs TAK1 inhibitor Cisplatin; ^#^ *P* < .05 vs Vehicle Cisplatin. n = 6 in each group. C, Representative Western blots show ERK protein expression in kidneys of vehicle group and TAK1 inhibitor group mice at 72 h after cisplatin or saline treatment. D, Quantitative analysis of protein expression ratio of phosphorylated ERK and total ERK in kidneys of mice. ***P < .001 vs Sham Vehicle; ^+^ *P* < .05 vs TAK1 inhibitor Cisplatin; ^#^ *P* < .05 vs Vehicle Cisplatin. n = 6 in each group

p38 and ERK are two main subfamilies of mammalian MAPKs, which are a group of serine/threonine‐specific protein kinases generally expressed in all cell types and are essential components of signal transduction in various physiological and pathological processes.^19^ Substantial evidence has demonstrated that p38 and ERK play important roles in regulating autophagy in the areas of acute or chronic tissue injury^20^, ^21^ To explore the mechanisms of cell autophagy responsible for the TAK1 signal pathway during cisplatin‐induced AKI, we examined whether TAK1 inhibition affects the phosphorylation of p38 and ERK. Our result showed that cisplatin‐induced AKI increased phosphorylation of p38 and ERK, as detected by Western blotting in kidneys of control group mice. The phosphorylation of p38 and ERK was significantly reduced in kidneys of TAK1 inhibitor group mice after cisplatin treatment (Figure 4A‐D). These data suggest that cisplatin activates TAK1, up‐regulating phosphorylation of p38 and ERK and subsequently mediates cell autophagy.

### 3.5 Inhibition of TAK1 decreases autophagy in renal tubular epithelial cells treated with cisplatin in vitro

**Figure 5:**
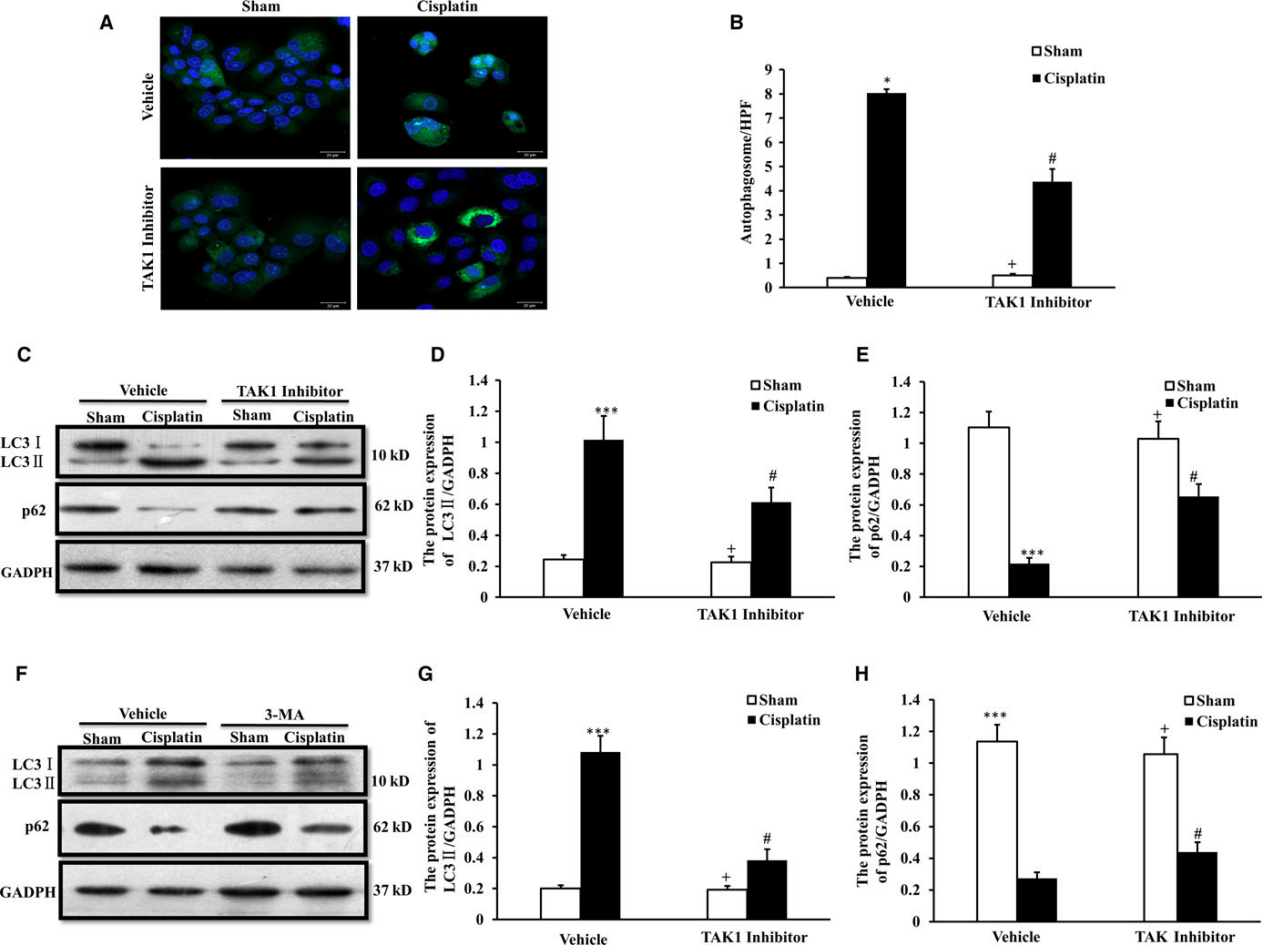
Inhibition of TAK1 decreases autophagy in renal tubular epithelial cells treated with cisplatin in vitro. A, GFP‐LC3 staining with vehicle and TAK1 inhibitor pre‐treated renal tubular epithelial cells treated with cisplatin (10 μg/mL, 6 h) or sham in vitro (Original magnification: ×100). B, Quantitative analysis of autophagosome with LC3 fluorescent spot (green). *P < .05 vs Sham Vehicle; ^+^ *P* < .05 vs TAK1 inhibitor Cisplatin; ^#^ *P* < .05 vs Vehicle Cisplatin. n = 4 in each group. C, Representative Western blots show LC3 I/II and p62 protein expression in vehicle and TAK1 inhibitor (5Z‐7‐oxozeaenol, 10 μmol/L, pre‐treated for 1 h) pre‐treated renal tubular epithelial cells treated with cisplatin (10 μg/mL, 6 h) or sham in vitro. D. Quantitative analysis of protein expression of LC3II in renal tubular epithelial cells. ***P < .001 vs Sham Vehicle; ^+^ *P* < .05 vs TAK1 inhibitor Cisplatin; ^#^ *P* < .05 vs Vehicle Cisplatin. n = 4 in each group. E. Quantitative analysis of protein expression of p62 in renal tubular epithelial cells. ***P < .001 vs Sham Vehicle; ^+^ *P* < .05 vs TAK1 inhibitor Cisplatin; ^#^ *P* < .05 vs Vehicle Cisplatin. n = 4 in each group. F. Representative Western blots show LC3 I/II and p62 protein expression in vehicle and 3‐MA (3‐MA, 10 μmol/L, pre‐treated for 1 h) pre‐treated renal tubular epithelial cells treated with cisplatin (10 μg/mL, 6 h) or sham in vitro. G, Quantitative analysis of protein expression of LC3II in renal tubular epithelial cells. ***P < .001 vs Sham Vehicle; ^+^ *P* < .05 vs 3‐MA Cisplatin; ^#^ *P* < .05 vs Vehicle Cisplatin. n = 4 in each group. H, Quantitative analysis of protein expression of p62 in renal tubular epithelial cells. ***P < .001 vs Sham Vehicle; ^+^ *P* < .05 vs 3‐MA Cisplatin; ^#^ *P* < .05 vs Vehicle Cisplatin. n = 4 in each group

To confirm if cisplatin activates TAK1 and subsequently mediates cell autophagy, we observed the autophagy level of renal tubular epithelial cells treated with cisplatin in vitro with GFP‐LC3 staining. Our results showed that the number of autophagosomes was markedly increased in renal tubular epithelial cells following cisplatin treatment. The number of autophagosomes was significantly reduced in renal tubular epithelial cells of the TAK1 inhibitor group compared with the vehicle control group following cisplatin treatment (Figure 5A,B).

We detected the expression of LC3 II and p62 in renal tubular epithelial cells following cisplatin treatment using the Western blot method (Figure 5C). The expression of LC3II was significantly higher in cells following cisplatin treatment compared with the sham control. The expression of LC3II was significantly decreased in cells of the TAK1 inhibitor group after cisplatin treatment compared with vehicle cells treated with cisplatin (Figure 5D). The level of p62 protein was markedly decreased in cells following cisplatin treatment compared with the sham control. The level of p62 protein was significantly increased in cisplatin‐treated cells of the TAK1 inhibitor group compared with vehicle cells treated with cisplatin (Figure 5E). The change trend of LC3II and p62 in renal tubular epithelial cells following 3‐MA treatment with Western blotting analysis was consistent with TAK1 inhibitor (Figure 5F‐H). These data indicate that inhibition of TAK1 decreases autophagy of renal tubular epithelial cells in response to cisplatin treatment.

### 3.6 Up‐regulation of TAK1 increases autophagy in renal tubular epithelial cells treated with cisplatin in vitro

**Figure 6:**
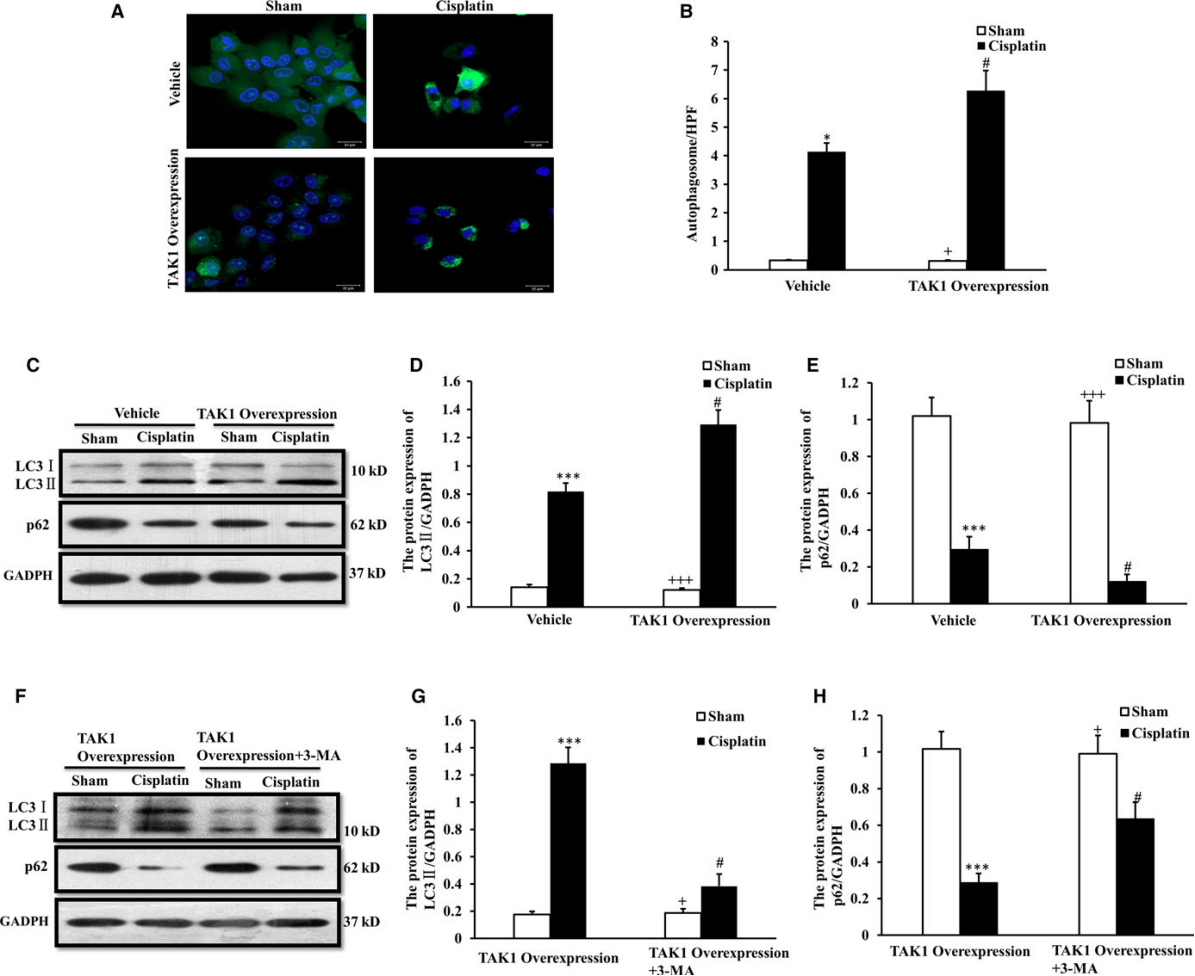
Up‐regulation of TAK1 increases autophagy in renal tubular epithelial cells treated with cisplatin in vitro. A, GFP‐LC3 staining with vehicle and TAK1 overexpression plasmid pre‐treated renal tubular epithelial cells treated with cisplatin (10 μg/mL, 6 h) or sham in vitro (Original magnification: ×100). B, Quantitative analysis of autophagosome with LC3 fluorescent spot (green). *P < .05 vs Sham Vehicle; ^+^ *P* < .05 vs TAK1 overexpression Cisplatin; ^#^ *P* < .05 vs Vehicle Cisplatin. n = 4 in each group. C, Representative Western blots show LC3 I/II and p62 protein expression in vehicle and TAK1 overexpression plasmid pre‐treated renal tubular epithelial cells treated with cisplatin (10 μg/mL, 6 h) or sham in vitro. D, Quantitative analysis of protein expression of LC3II in renal tubular epithelial cells. ***P < .001 vs Sham Vehicle; ^+++^ *P* < .001 vs TAK1 overexpression Cisplatin; ^#^ *P* < .05 vs Vehicle Cisplatin. n = 4 in each group. E, Quantitative analysis of protein expression of p62 in renal tubular epithelial cells. ***P < .001 vs Sham Vehicle; ^+++^ *P* < .001 vs TAK1 overexpression Cisplatin; ^#^ *P* < .05 vs Vehicle Cisplatin. n = 4 in each group. F, Representative Western blots show LC3 I/II and p62 protein expression in TAK1 overexpression plasmid and TAK1 overexpression plasmid plus 3‐MA (10 μmol/L) pre‐treated renal tubular epithelial cells treated with cisplatin (10 μg/mL, 6 h) or sham in vitro. G, Quantitative analysis of protein expression of LC3II in renal tubular epithelial cells. ***P < .001 vs Sham Vehicle; ^+^ *P* < .05 vs TAK1 overexpression Cisplatin; ^#^ *P* < .05 vs Vehicle Cisplatin. n = 4 in each group. H, Quantitative analysis of protein expression of p62 in renal tubular epithelial cells. ***P < .001 vs TAK1 overexpression Sham; ^+^ *P* < .05 vs TAK1 overexpression plus 3‐MA Cisplatin; ^#^ *P* < .05 vs TAK1 overexpression Cisplatin. n = 4 in each group

Then, we applied a TAK1 overexpression vector to reverse authenticate the effect of TAK1 on cell autophagy using renal tubular epithelial cells treated with cisplatin in vitro. Our results showed that the number of autophagosomes was significantly increased in renal tubular epithelial cell pre‐processing with TAK1 overexpression compared with vehicle control cells following cisplatin treatment (Figure 6A,B). The expression of LC3B and p62 in renal tubular epithelial cell pre‐processing with TAK1 overexpression or vehicle following cisplatin treatment was assessed by the Western blot method (Figure 6C). The results of the ratio of LC3B II/I and the level of p62 protein indicated that up‐regulation of TAK1 increases autophagy in renal tubular epithelial cells treated with cisplatin (Figure 6D,E). The expression of LC3B and p62 in renal tubular epithelial cell pre‐processing with TAK1 overexpression or TAK1 overexpression combinated with 3‐MA following cisplatin treatment was assessed by the Western blot method (Figure 6F). The results of the ratio of LC3B II/I and the level of p62 protein indicated that 3‐MA decreased autophagy induced by TAK1 up‐regulation in renal tubular epithelial cells treated with cisplatin (Figure 6G,H). These data indicate that up‐regulation of TAK1 increases autophagy in renal tubular epithelial cells treated with cisplatin.

### 3.7 TAK1 mediates phosphorylation of p38 and ERK to increase autophagy of renal tubular epithelial cells treated with cisplatin in vitro

**Figure 7:**
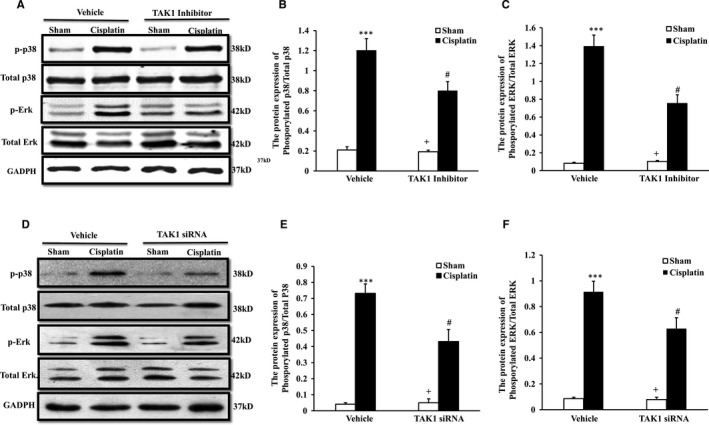
Inhibition of TAK1 or TAK1 knock‐down decreases p38 and ERK phosphorylation of renal tubular epithelial cells with cisplatin treatment. A, Representative Western blots showed phosphorylation levels of p38 and ERK in vehicle or TAK1 inhibitor pre‐treated renal tubular epithelial cells of mice with cisplatin 10 μg/mL or sham treatment 6 h. B, Quantitative analysis of p38 phosphorylation in vehicle or TAK1 inhibitor pre‐treated renal tubular epithelial cells with cisplatin or sham treatment. ***P < .001 vs Sham Vehicle; ^+^ *P* < .05 vs TAK1 inhibitor Cisplatin; ^#^ *P* < .05 vs Vehicle Cisplatin. n = 4 in each group. C, Quantitative analysis of ERK phosphorylation in vehicle or TAK1 inhibitor pre‐treated renal tubular epithelial cells with cisplatin or sham treatment. ***P < .001 vs Sham Vehicle; ^+^ *P* < .05 vs TAK1 inhibitor Cisplatin; ^#^ *P* < .05 vs Vehicle Cisplatin. n = 4 in each group. D, Representative Western blots showed phosphorylation levels of p38 and ERK in vehicle or TAK1 siRNA pre‐treated renal tubular epithelial cells of mice with cisplatin (10 μg/mL, 6 h) or sham in vitro. E, Quantitative analysis of p38 phosphorylation. ***P < .001 vs Vehicle Sham; ^+^ *P* < .05 vs TAK1 siRNA Cisplatin; ^#^ *P* < .05 vs Vehicle Cisplatin. n = 4 in each group. F, Quantitative analysis of ERK phosphorylation. ***P < .001 vs Vehicle Sham; ^+^ *P* < .05 vs TAK1 siRNA Cisplatin; ^#^ *P* < .05 vs Vehicle Cisplatin. n = 4 in each group

To verify if TAK1 mediates p38 and ERK signalling pathways to regulate autophagy of the renal tubular epithelial cells responsible for cisplatin treatment, we,respectively, examined the phosphorylation of p38 and ERK of renal tubular epithelial cells treated with TAK1 inhibitor plus cisplatin (Figure 7A) and TAK1 siRNA plus cisplatin (Figure 7D) in vitro via the Western blotting method. Our result showed that cisplatin treatment significantly increased phosphorylation of p38 and ERK in renal tubular epithelial cells treated with cisplatin. The phosphorylation of p38 and ERK was significantly reduced in renal tubular epithelial cells with TAK1 inhibitor preconditioning after cisplatin treatment (Figure 7B,C). The change trend of p38 and ERK in renal tubular epithelial cells following TAK1 siRNA treatment with Western blotting analysis was consistent with TAK1 inhibitor (Figure 7E,F).

**Figure 8:**
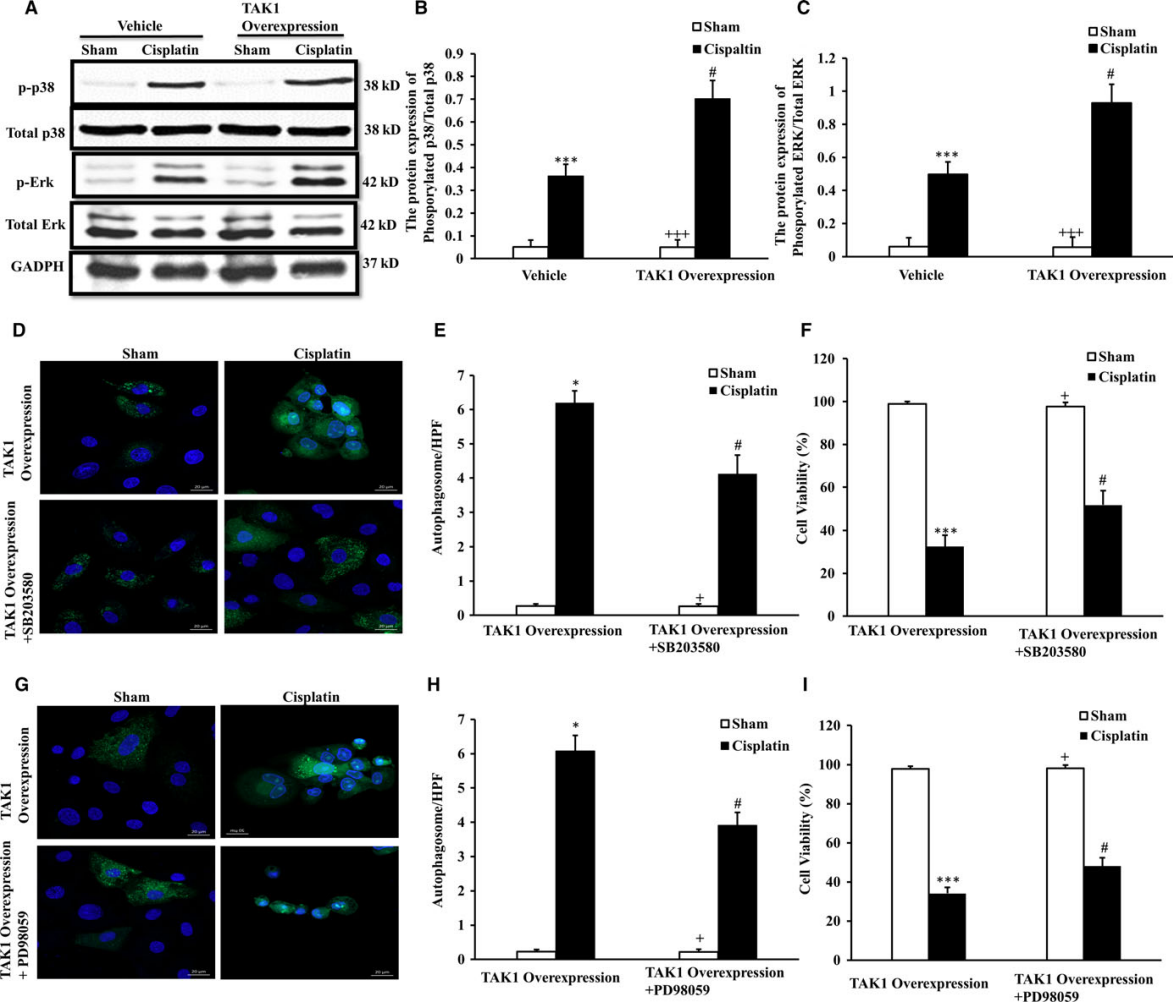
Up‐regulation of TAK1 increases p38 and ERK phosphorylation of renal tubular epithelial cells with cisplatin treatment. A, Representative Western blots showed phosphorylation levels of p38 and ERK in vehicle or TAK1 overexpression plasmid pre‐treated renal tubular epithelial cells of mice with cisplatin (10 μg/mL, 6 h) or sham in vitro. B, Quantitative analysis of p38 phosphorylation. ***P < .001 vs Sham Vehicle; ^+++^ *P* < .001 vs TAK1 overexpression Cisplatin; ^#^ *P* < .05 vs Vehicle Cisplatin. n = 4 in each group. C, Quantitative analysis of ERK phosphorylation. ***P < .001 vs Sham Vehicle; ^+++^ *P* < .001 vs TAK1 overexpression Cisplatin; ^#^ *P* < .05 vs Vehicle Cisplatin. n = 4 in each group. D, GFP‐LC3 staining with TAK1 overexpression plasmid or TAK1 overexpression plasmid plus SB203580 pre‐treated renal tubular epithelial cells treated with cisplatin (10 μg/mL, 6 h) or sham in vitro (Original magnification: ×100). E, Quantitative analysis of autophagosome with LC3 fluorescent spot (green). *P < .05 vs TAK1 overexpression Sham; ^+^ *P* < .05 vs TAK1 overexpression+ SB203580 Cisplatin; ^#^ *P* < .05 vs TAK1 overexpression Cisplatin. n = 4 in each group. F, Quantitative analysis of cells viability with TAK1 overexpression plasmid or TAK1 overexpression plasmid plus SB203580 pre‐treated renal tubular epithelial cells treated with cisplatin or sham in vitro. ***P < .001 vs TAK1 overexpression Sham; ^+^ *P* < .05 vs TAK1 overexpression+ SB203580 Cisplatin; ^#^ *P* < .05 vs TAK1 overexpression Cisplatin. n = 4 in each group. G, GFP‐LC3 staining with TAK1 overexpression plasmid or TAK1 overexpression plasmid plus PD98059 pre‐treated renal tubular epithelial cells treated with cisplatin (10 μg/mL, 6 h) or sham in vitro (Original magnification: ×100). H, Quantitative analysis of autophagosome with LC3 fluorescent spot (green). *P < .05 vs TAK1 overexpression Sham; ^+^ *P* < .05 vs TAK1 overexpression + PD98059 Cisplatin; ^#^ *P* < .05 vs TAK1 overexpression Cisplatin. n = 4 in each group. I, Quantitative analysis of cells viability with TAK1 overexpression plasmid or TAK1 overexpression plasmid plus PD98059 pre‐treated renal tubular epithelial cells treated with cisplatin or sham in vitro. ***P < .001 vs TAK1 overexpression Sham; ^+^ *P* < .05 vs TAK1 overexpression + PD98059 Cisplatin; ^#^ *P* < .05 vs TAK1 overexpression Cisplatin. n = 4 in each group

We applied a TAK1 overexpression vector to reverse verify the effect on the p38 and ERK signalling pathways with up‐regulation of TAK1 using renal tubular epithelial cells treated with cisplatin in vitro with Western blotting analysis (Figure 8A). Our result showed that the phosphorylation of p38 and ERK was significantly further increased in renal tubular epithelial cells with TAK1 overexpression vector preconditioning after cisplatin treatment (Figure 8B,C).

To confirm their role of p38 or ERK in the regulation of autophagy and AKI in particular in the direct relationship with TAK1 overexpression, we applied TAK1 overexpression vector applied TAK1 overexpression vector plus p38 inhibitor (Figure 8D) or ERK inhibitor (Figure 8G) respectively to reverse authenticate the effect of p38 or ERK on cell autophagy induced by TAK1 using renal tubular epithelial cells treated with cisplatin in vitro with GFP‐LC3 staining. Our results showed that the number of autophagosomes was significantly decreased in renal tubular epithelial cell pre‐processing with TAK1 overexpression vector combinated p38 inhibitor (Figure 8E) or ERK inhibitor (Figure 8H) compared with TAK1 overexpression vector control cells following cisplatin treatment. Then, to investigate the effect on injury with autophagy mediated with the pathways of TAK1/p38 or TAK1/ERK in vitro, the cells viability was detected with MTT colorimetric assay using TAK1 overexpression vector combinated with p38 inhibitor (Figure 8F) or ERK inhibitor (Figure 8I) in the model of renal tubular epithelial cells treated with cisplatin. These data indicate that TAK1 mediated with p38 and ERK increases autophagy to aggravate cisplatin‐induced injury.

## 4 DISCUSSION

Cisplatin is one of the most common cancer drugs in clinical application, with a wide anticancer spectrum, strong penetrating power, high curative effect and synergy with a variety of antitumour drug.^22^ Nevertheless, the serious renal toxicity of cisplatin limits its clinical application.^23^ The mechanism of renal damage induced by cisplatin is still unclear. TAK1 is located upstream of MAPK and IKB kinases.^24^ A significant body of evidence suggests that the TGF‐β signalling pathway mediates acute renal injury^25^, ^26^ and that p38 and ERK, as members of the MAPK family, play a crucial role in autophagy.^20^ Therefore, we hypothesized that TAK1 might regulate the p38 and ERK signalling pathways to mediate autophagy involved in the mechanism of cisplatin‐induced AKI. Experiments in vivo and vitro in our study implicated that TAK1 is activated in cisplatin‐induced AKI; inhibition of TAK1 alleviated damage of cisplatin‐induced AKI and autophagy of renal tubular epithelial cells; and TAK1 regulates p38 and ERK signalling pathways in the pathogenesis of cisplatin‐induced AKI. Our results indicate that TAK1 mediates excessive autophagy via p38 and ERK signalling pathways in the mechanism of cisplatin‐induced AKI.

TAK1 is an important signal transducer within cells and is essentially a serine/threonine kinase. The biological effects of TAK1 are involved in almost all aspects of cell function, including cell proliferation, differentiation, survival, apoptosis, autophagy and the inflammatory immune response.^27^ Increasing evidence has manifested that TAK1 is widely expressed in multiple important organs and is involved in the pathophysiological processes of acute injury by regulating and mediating multiple signalling pathways^7^, ^28^ In our previous study, we demonstrated that TAK1 mediated apoptosis in ischaemia‐reperfusion of the kidney.^29^ Ma FY found that TAK1 regulates inflammation and fibrosis in the obstructed kidney.^30^ However, it is unclear if TAK1 plays a regulated role in cisplatin‐induced AKI and, if so, its molecular mechanism. In the present work, we have detected that the expression level of TAK1 was increased in the kidney of mice treated with cisplatin. Then, we examined the role of TAK1 in the kidney of mice following cisplatin‐induced AKI using a specific TAK1 inhibitor. Our study shows that inhibition of TAK1 significantly reduces tubular damage in the kidney of mice following cisplatin treatment. These data strongly support an important role of TAK1 in the development of cisplatin‐induced AKI.

Some evidence indicated that autophagy plays a protective role in diverse pathologies, including infection, cancer, acute and chronic injury, neurodegeneration, ageing and other diseases in the past years. However, emerging evidence indicated that the self‐cannibalistic function may be associated with excessive cell death under certain conditions^31^, ^32^ Therefore, more and more scholars described the role of autophagy in various pathophysiological processes as a double‐edged sword.^33^, ^34^, ^35^, ^36^ From the previous study, the autophagy was proved to be a protective role for AKI^37^, ^38^^.^However, Chien, Suzuki, Inoue and colleagues suggested the role of autophagy in tubular cell death, which are consistent with our results.^39^, ^40^, ^41^ Recent evidence also suggests that autophagy promotes the development of cellular senescence, which contributes to renal ageing and promotes the progression from AKI to CKD.^42^ Such controversy also proves that autophagy plays a double‐edged sword in the pathogenesis of AKI. Different trail methods and conditions, including stimulation methods, drug dosage, the duration of action would achieve the double‐sided results of autophagy. Even in the study with Jiang M,^37^ they also found that autophagy indicators were significantly reduced in 3 days after cisplatin treatment. Moreover, in keeping with the dual anti‐inflammatory and pro‐inflammatory capabilities of autophagy as an innate immunity mechanism, basal autophagy keeps inflammasomes from being spuriously activated by intracellular triggers, but induced autophagy can augment inflammasome‐dependent processing, which would be aggravated AKI.^43^ Finally, the downstream signal pathway of TAK1 is complicated and interacted,^44^ and the autophagy is also affected by cell transfection and inhibitor of signal pathways. All of these factors may affect the ultimate effect of autophagy in the pathogenesis of cisplatin‐induced AKI.

It is worth mentioning that apoptosis contributed to excessive cell death in AKI is generally determined and some researchers indicated that apoptosis and autophagy are co‐ordinated processes in the pathogenesis of AKI recently.^42^ The dual role of autophagy under stimulating factor involves complicated crosstalk of autophagy and apoptosis. Autophagy can not only block the induction of apoptosis, but also help to induce apoptosis. Moreover, the interactions of different autophagy‐ and apoptosis‐related proteins also have common up‐ or downstream signalling pathways, which make the mutual regulatory mechanisms between apoptosis and autophagy remain complex. Generally, autophagy blocks the induction of apoptosis and inhibits the activation of apoptosis‐associated caspase which could reduce cellular injury.^45^ However, in special cases, autophagy or autophagy‐relevant proteins may help to induce apoptosis, which could aggravate cell damage.^46^ Therefore, it is not surprising that many scholars have drawn opposite conclusions about the role of autophagy in various acute injuries.

MAPKs play an important role in biological processes, including various types of cell death.^47^ A large amount of evidence have indicated that p38 and ERK, the two important MAPK family members, are important upstream molecules in regulating mTOR activity and are necessary for the induction of autophagy.^48^, ^49^ vom Dahl et al demonstrated that p38 played a key role in cell swelling‐induced autophagy, and a specific inhibitor of p38 strongly decreased the volume of autophagosomes.^50^ The research from Ponnusamy et al points out that specific inhibition of the ERK pathway could block autophagy in the pathogenesis of renal fibroblasts.^51^ Furthermore, TAK1 is an important signalling pathway located upstream of p38 and ERK, which play critical roles in various types of physiological processes^52^, ^53^^.^Kim SI showed that TGF‐β1 induced autophagy in mesangial cells via the TAK1‐MKK3‐p38 signalling pathway.^54^ TAK1 is upstream of ERK and regulates the TNF‐α‐mediated activation of NF‐κB, which is associated with inflammation.^55^ Our data show that inhibition of TAK1 significantly decreased the levels of phosphorylated p38 and ERK with treatment of cisplatin in vivo and in vitro, which were all markedly increased with cisplatin treatment. In addition, up‐regulation of TAK1 with an overexpression vector reversely verified that the p38 and ERK signalling pathways were regulated by TAK1 in cisplatin‐induced AKI. Combining the accepted theories that p38 and ERK are the positive regulatory signalling pathway of autophagy, we have enough reasons to conclude that TAK1 mediates phosphorylation of p38 and ERK, leading to excessive autophagy of tubular epithelial cells and exacerbated kidney damage in cisplatin‐induced AKI.

In summary, our study defines a novel mechanism of TAK1 participation in cisplatin‐induced AKI. In response to stimulation with cisplatin, activation of TAK1 leads to p38 and ERK phosphorylation and then to excessive autophagy, which plays a critical role in the pathogenesis of cisplatin‐induced AKI.

## CONFLICT OF INTEREST

The authors declared that they have no conflict of interest to this work. We declare that we do not have any commercial or associative interest that represents a conflict of interest in connection with the work submitted.
