# Correction to “TAK1 mediates excessive autophagy via p38 and ERK in cisplatin‐induced acute kidney injury”

**DOI:** 10.1111/jcmm.70414

**Published:** 2025-02-12

Zhou J, Fan Y, Zhong J, et al. TAK1 mediates excessive autophagy via p38 and ERK in cisplatin‐induced acute kidney injury. *Journal of cellular and molecular medicine* 2018;22(5):2908–2921. doi:10.1111/jcmm.13585.

In Zhou Jun et al., the immunohistochemistry (IHC) image for sham in Figure 1H was inadvertently misused during the figure preparation process. This correction does not affect the figure legends or the conclusions drawn in the study. The correct figures are shown below.

**FIGURE 1:**
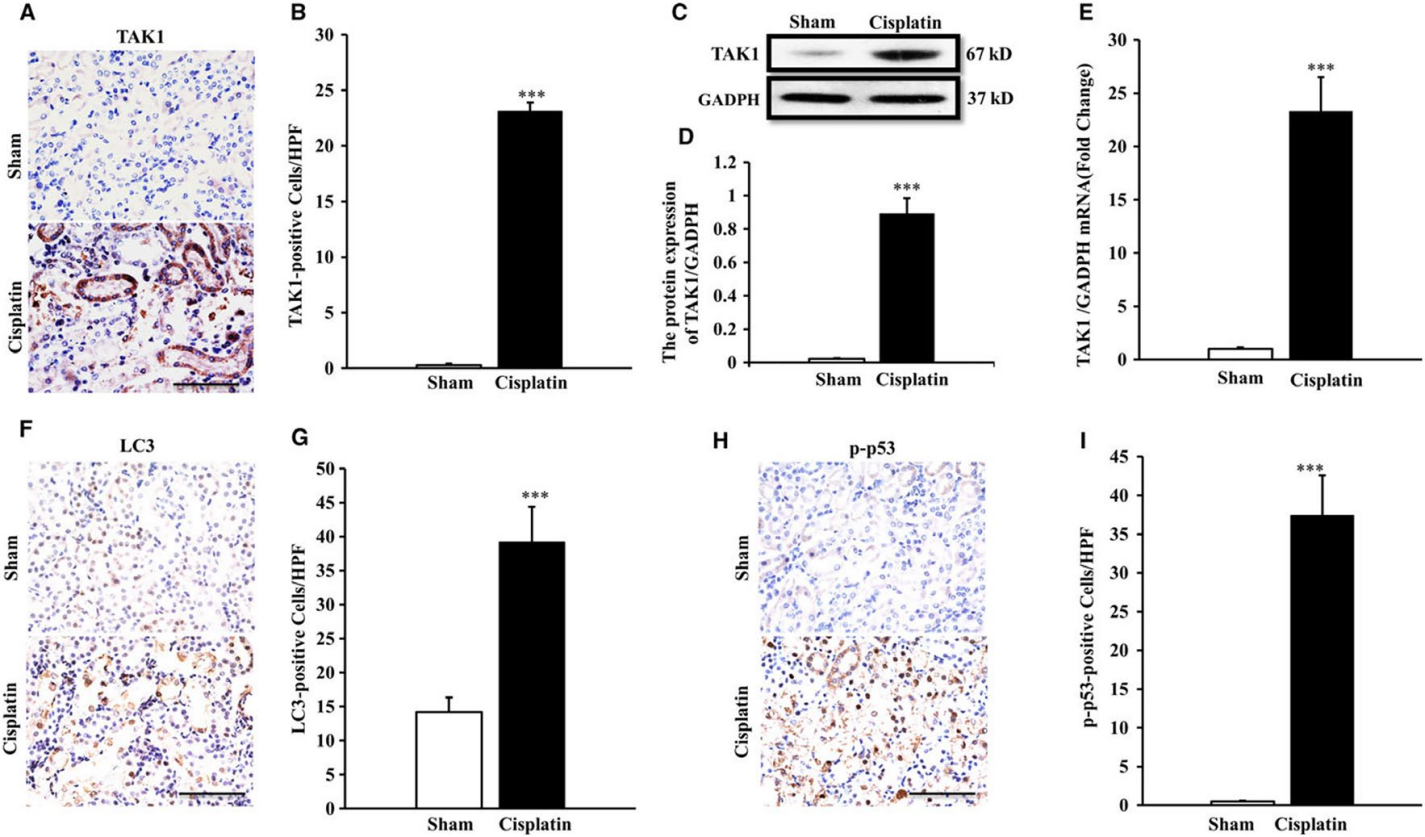
TAK1 expression in kidneys of mice with cisplatin‐induced AKI. (A) Representative photomicrographs of TAK1 immunohistochemical staining in kidneys of mice ip with cisplatin (20 mg/kg) or saline (original magnification: 9400, scale bar: 50 lm). (B) Quantitative analysis of TAK1‐positive cells in kidneys of mice ip with cisplatin (20 mg/kg) or saline. ***p < 0.001 versus Sham, *n* = 6 each. (C) Representative western blots show TAK1 protein expression in kidneys of mice after sham or cisplatin treatment. (D) Quantitative analysis of protein expression of TAK1 in kidneys of mice. ***p < 0.001 vs. Sham, *n* = 6 in each group. (E) Quantitative analysis of TAK1 mRNA in kidneys of mice. ***p < 0.001 versus Sham, *n* = 6 in each group. (F) Representative photomicrographs of LC3 immunohistochemical staining in kidneys of mice ip with cisplatin (20 mg/kg) or saline (original magnification: 9400, scale bar: 50 lm). (G) Quantitative analysis of LC3‐positive cells in kidneys of mice. ***p < 0.001 vs. Sham, *n* = 6 each. (H) Representative photomicrographs of p‐p53 immunohistochemical staining in kidneys of mice ip with cisplatin (20 mg/kg) or saline (original magnification: 9400, scale bar: 50 lm). (I) Quantitative analysis of p‐p53‐positive cells in kidneys of mice. ***p < 0.001 vs. Sham, *n* = 6 each.

